# The Mobile Health Multiple Lifestyle Behavior Interventions Across the Lifespan (MoBILE) Research Program: Protocol for Development, Evaluation, and Implementation

**DOI:** 10.2196/14894

**Published:** 2020-04-20

**Authors:** Marcus Bendtsen, Preben Bendtsen, Hanna Henriksson, Pontus Henriksson, Ulrika Müssener, Kristin Thomas, Marie Löf

**Affiliations:** 1 Department of Health, Medicine and Caring Sciences Linköping University Linköping Sweden; 2 Department of Medical Specialist Motala Sweden; 3 Department of Biosciences and Nutrition Karolinska Institutet Stockholm Sweden

**Keywords:** telemedicine, mHealth, eHealth, life style, randomized controlled trial, focus groups

## Abstract

**Background:**

Clustering of multiple lifestyle risk behaviors has been associated with a greater risk of noncommunicable diseases and mortality than one lifestyle risk behavior or no lifestyle risk behaviors. The National Board of Health and Welfare in Sweden reported in 2018 that it is important to provide additional support to individuals with multiple lifestyle risk behaviors, as risks from these behaviors are multiplicative rather than additive. However, the same report emphasized that there is a lack of knowledge regarding interventions that support changes to unhealthy lifestyle behaviors.

**Objective:**

The MoBILE (Mobile health Multiple lifestyle Behavior Interventions across the LifEspan) research program has brought together two Swedish research groups supported by international collaborators. Through this collaboration, we aim to design and evaluate a number of novel and tailored mobile health (mHealth) multiple lifestyle behavior interventions across the life span of different health care populations. In addition, the MoBILE research program will extend ongoing research to include mHealth interventions for migrant pregnant women and children.

**Methods:**

Each project within the MoBILE program will focus on a specific group: pregnant women, preschool children, high school and university students, and adults in primary and clinical care. All the projects will follow the same 4 phases: requirements, development, evaluation, and implementation. During the requirements phase, implementers and end users will aid the design of content and functionality of the interventions. In the development phase, findings from the first phase will be synthesized with expert domain knowledge and theoretical constructs to create interventions tailored to the target groups. The third phase, evaluation, will comprise randomized controlled trials conducted to estimate the effects of the interventions on multiple lifestyle risk behaviors (eg, alcohol, nutrition, physical activity, and smoking). The final phase will investigate how the interventions, if found effective, can be disseminated into different health care contexts.

**Results:**

The research program commenced in 2019, and the first results will be available in 2020. Projects involving pregnant women, preschool children, and high school and university students will be completed in the first 3 years, with the remaining projects being planned for the program’s final 3 years.

**Conclusions:**

The development of evidence-based digital tools is complex, as they should be guided by theoretical frameworks, and requires large interdisciplinary teams with competence in technology, behavioral science, and lifestyle-specific areas. Individual researchers or smaller research groups developing their own tools is not the way forward, as it means reinventing the wheel over and over again. The MoBILE research program therefore aims to join forces and learn from the past 10 years of mHealth research to maximize scientific outcomes, as well as the use of financial resources to expand the growing body of evidence for mHealth lifestyle behavior interventions.

**International Registered Report Identifier (IRRID):**

PRR1-10.2196/14894

## Introduction

### Background

An unhealthy diet, physical inactivity, smoking, and excessive consumption of alcohol are well-established risk factors for noncommunicable diseases (NCDs), such as cardiovascular disease, cancer, respiratory disease, and type 2 diabetes [1]. The Global Burden of Disease study from 2015 [2] reported that NCDs are responsible for 70% of the deaths globally each year, and according to the World Health Organization (WHO), the total annual number of deaths from NCDs will increase to 55 million by 2030 if the trend is not reversed.

Clustering of multiple lifestyle risk behaviors has been associated with a greater risk of NCDs and mortality compared with one lifestyle risk behavior or no lifestyle risk behaviors [3]. The National Board of Health and Welfare in Sweden reported in 2018 [4] that it is important to provide additional support to individuals with multiple lifestyle risk behaviors, as risks from multiple unhealthy lifestyle behaviors are multiplicative rather than additive. However, the same report emphasized that there is a lack of knowledge regarding interventions that support changes to unhealthy lifestyle behaviors.

WHO has published a global action plan for 2013 to 2020 to reduce NCDs, with one of the objectives being to strengthen health care systems to improve prevention and self-management of people with, or at high risk for, cardiovascular disease, cancer, chronic respiratory disease, and type 2 diabetes [5]. One of the arching principles in the plan is a life-course approach. This is essential as unhealthy lifestyle behaviors tend to be established in early childhood and adolescence, tracking into adulthood [6,7]. Therefore, an effort should be made to establish evidence-based lifestyle interventions both for those who are already experiencing the negative consequences of their unhealthy lifestyle behavior as well as those in life’s early stages, before risk behaviors lead to negative consequences.

### Electronic Health and Mobile Health: Large Potential for Lifestyle Interventions and Disease Management in Health Care

Globally, mobile phone subscriptions increased by 97% between 2000 and 2015, with 95% of the population (7 billion people) residing in an area with a mobile cellular network [8]. The Swedish population is described as one among the most digitally mature in the world [9], with high mobile phone ownership (97%) [10]. This digitalization has radically changed communication in areas such as the travel industry and banking sector; however, it also offers great opportunities for the future of health care.

Electronic health (eHealth) has been defined by WHO as the use of information and communication technologies for health. This broad definition encapsulates electronic patient records, patient management systems, lab systems, big data, and informatics. An additional component of eHealth is mobile health (mHealth), which has been defined as a medical or public health practice that is supported by mobile devices [11]. Mobile devices include mobile phones, patient monitoring devices, and other wireless devices.

In the past 10 to 15 years, there has been a strong increase in research investigating mHealth intervention programs for improving modifiable risk factors for chronic diseases, including diet, physical activity, weight loss, smoking cessation, and alcohol consumption reduction [12-17]. mHealth has also been used for disease management to assist, inform, guide, and treat patients with various acute and chronic diseases and disorders, such as type 2 diabetes, cardiovascular disease, and mental illness [18,19]. Some of the potential benefits of using mHealth interventions instead of more traditional face-to-face interventions or disease management programs are as follows: they can be delivered at any time or place, participants do not have to visit a clinic (or the number of visits can be reduced), the programs are interactive, and they can more easily be tailored toward specific groups.

### The Mobile Health Multiple Lifestyle Behavior Interventions Across the LifEspan Research Program

mHealth interventions offer new potential for the delivery of interventions that promote healthy lifestyles and self-management of NCDs, and the interest for developing such interventions has rapidly increased. However, instead of reinventing the wheel, it is time to join forces and learn from the past 10 years of mHealth research to maximize scientific outcomes and financial resources to move this research area forward.

The MoBILE (Mobile health Multiple lifestyle Behavior Interventions across the LifEspan) research program has brought together two strong Swedish research groups supported by international collaborators: The Innovative use of mobile phones to promote physical activity and nutrition across the lifespan (IMPACT) research group and the Lifestyle Intervention Implementation Research (LiiR) group.

The IMPACT group at Karolinska Institutet and Linköping University (led by Professor Marie Löf) has expertise in nutrition, physical activity, and behavioral science [20-24]. This group has more than 10 years of experience in developing mHealth interventions, with special emphasis on pregnant women and young children [25-27]. The LiiR group at Linköping University (led by Senior Lecturer Marcus Bendtsen) has expertise in medicine, occupational therapy, health psychology, and computer science and statistics. This group has nearly 15 years of experience in developing eHealth and mHealth interventions focusing on smoking cessation, alcohol consumption, physical activity, and positive psychology [28-41]. The group has also conducted research on how to implement such interventions into daily routine in primary health care, universities, high schools, and workplaces [30,36,41-43].

This interdisciplinary cooperation includes investigators with expertise in the big 4 lifestyle risk behaviors (ie, diet, physical activity, smoking, and alcohol), behavioral science, medicine, statistics, machine learning, and information technology. Through such collaboration, we will design and evaluate a number of novel and tailored mHealth multiple lifestyle behavior interventions across the life span of different health care populations. The research program also aims to establish an excellence research center where knowledge and expertise will be disseminated to the scientific community, health professionals, and stakeholders in the eHealth and mHealth area. The program has received a 3-year grant, extendable to 6 years, from the Swedish Research Council for Health, Working Life, and Welfare (FORTE, Dnr: 2018-01410).

The motivation behind the MoBILE research program can be described by using 3 major research gaps in the field. First, we need additional evidence to decide whether multiple lifestyle behavior interventions are effective. Second, to help more individuals, we need to better understand what works and for whom, with respect to different content and functionality of mHealth interventions. Third, we need to understand how interventions can be made accessible to everyone in the community, with a special emphasis on individuals with a migrant background. The following sections provide a detailed explanation of each research gap.

#### Research Gap 1: Are Multiple Lifestyle Behavior Interventions Through Mobile Health the Way Forward?

Since the early 2000s, there has been great interest in multiple lifestyle behavior interventions [44]. Simultaneously addressing multiple lifestyle risk behaviors in interventions may result in increased confidence in the capacity for change and enhance effectiveness. Indeed, there is some evidence supporting that interventions targeting multiple lifestyle risk behaviors at the same time may be beneficial for improving the general lifestyle of individuals [45,46]. However, two recent meta-analyses reported modest effects regarding multiple lifestyle risk behaviors in nonclinical [44] and clinical populations [47]. Various reasons were suggested, including poor implementation of the intervention. Implementation difficulties may be hard to overcome with traditional delivery modes, such as face-to-face interventions, because of the huge demand of resources such as time and staff.

Therefore, mHealth offers new potential to this research area, because of its ability to work autonomously from health care professionals and as interventions that are digital are more flexible with respect to content and delivery than face-to-face and other traditional means of content delivery. However, in a meta-analysis of multiple lifestyle risk behaviors from 2017, only 4 of the 69 randomized controlled trials utilized eHealth technology as the sole delivery mode (eg, email, SMS text message, or website) [44]. In addition, these 4 trials were limited by low power or engagement or had questionable external validity.

In summary, mHealth provides new potential to achieve changes in unhealthy lifestyle behaviors; however, to date, the potential of mHealth interventions in this area has been scarcely explored. The MoBILE research program will utilize the complementary expertise in all 4 lifestyle behaviors and mHealth, gained by combining the efforts of research groups, to design and evaluate 7 mHealth multiple lifestyle behavior interventions across the life span of different health care populations.

#### Research Gap 2: What Works and for Whom in Mobile Health Interventions?

Although an increasing number of mHealth interventions are being developed for prevention and management of chronic diseases, there is a knowledge gap on how to best develop and implement such interventions, taking both patients’ and health care staffs’ views into consideration [48,49]. For instance, patients might prefer visiting the health care clinic in person rather than monitoring their health at home; on the other hand, the staff might expect a reduction in their workload by patients using digital tools, which might not always be the case [50]. Thus, a key area is how to motivate both patients and staff to engage in the effective use of mHealth interventions [51].

Therefore, it is important to apply a user-centered approach when developing digital interventions; however, the consideration for the implementers’ expectations and readiness to implement the suggested interventions must also be taken into account [52]. The MoBILE research program will address this research gap by including both end users and implementers in the development of 7 mHealth lifestyle behavior interventions, taking into account their respective requirements and expectations.

#### Research Gap 3: How Do We Make Mobile Health Interventions Accessible for All?

Low socioeconomic status and migrant background are associated with inferior health; however, mobile phones are commonly accessible, irrespective of the socioeconomic status [53]. In addition, mHealth solutions can offer flexibility in terms of content, level of information (ie, advanced text, easy-to-read text, or pictures), and languages. Thus, mHealth also offers the potential to reach groups that are hard to reach with traditional face-to-face interventions in health care. However, the use of mHealth in interventions to promote healthy lifestyle behaviors among socially disadvantaged groups and migrant populations is sparse but growing rapidly. Mostly, pilot studies have been conducted thus far; however, several studies have reported promising results [54,55].

Key target groups in the Swedish context include pregnant women and young children. Approximately 25% of the women attending maternity clinics are born outside of Sweden. Thus, mHealth tools to promote a healthy lifestyle among pregnant women and their children should also be accessible to migrant populations. Consequently, a key area for research in the mHealth area is to tailor content and features to these populations.

The MoBILE research program will extend ongoing research to also include mHealth interventions for migrant pregnant women and children, as well as build on this work for other populations in the program.

### Specific Aims

The overall purpose of the MoBILE research program is to design, evaluate, and implement 7 state-of-the-art mHealth multiple lifestyle behavior interventions, which can be promoted by health care professionals. These are self-management interventions that aim to support a healthy lifestyle across the life span of different populations. Specifically, the program will be built around the following aims:

1. To investigate user requirements (patients and health care providers) for 7 mHealth multiple lifestyle behavior interventions in terms of technology and content in different health care populations (research gaps 2 and 3).
2. To assess the effectiveness of 7 mHealth multiple lifestyle behavior interventions in different health care populations (research gap 1).
3. To set a standard for how predictive, rather than explanatory, statistical modelling can be used for investigating who benefits from which multiple lifestyle behavior intervention in different health care populations (research gap 1).
4. To assess, using causal inference, the mediating effects of a number of mHealth multiple lifestyle behavior interventions through psychological factors, such as self-efficacy and motivation, in different health care populations (research gap 1).
5. To evaluate the extent to which the effectiveness, engagement, and user satisfaction for the specific mHealth tools differ among end users with different socioeconomic status and migrant backgrounds in different health care populations (research gap 3).
6. To tailor mHealth multiple lifestyle behavior interventions to groups with a migrant background in 2 health care populations (ie, pregnant women and preschool children) and to build on the knowledge gained to modify the other interventions in the program when relevant (research gap 3).
7. To implement the aforementioned mHealth multiple lifestyle behavior interventions that are deemed effective and to evaluate how well they are adopted by routine health care (research gaps 1 and 2).

## Methods

### Overview

**Figure 1:**
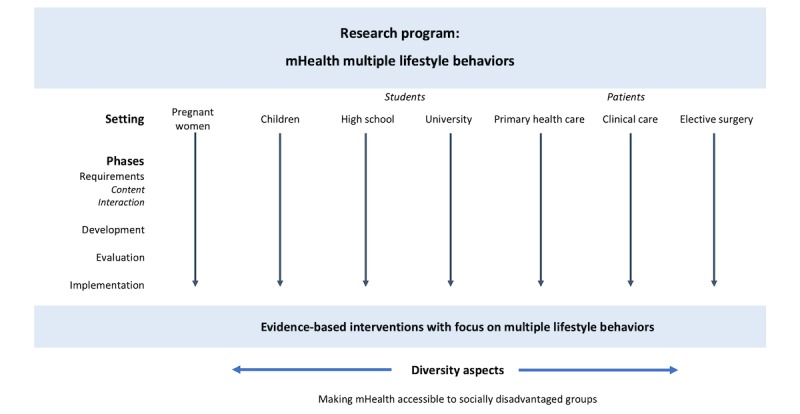
Schematic presentation of the Mobile health Multiple lifestyle Behavior Interventions across the LifEspan research program. mHealth: mobile health.

A schematic presentation of the MoBILE research program is provided in Figure 1. It covers both the primary care setting as well as the specialized clinical setting and includes 7 mHealth multiple lifestyle behavior intervention projects. Each project will focus across the life span of a specific group: pregnant women, preschool children, high school and university students, and adults in primary and clinical care. All the projects will follow the same 4 phases: requirements, development, evaluation, and implementation. The rest of this section is laid out as follows: first, we introduce each of the 4 phases, which is common for all projects; thereafter, we briefly discuss the details of each project. Note that in preparation for each project, trial registration and protocols will be made available with full details before trial commencement, including recruitment and statistical analysis plans.

### The Phases of the Mobile Health Multiple Lifestyle Behavior Interventions Across the Lifespan Research Program

#### Phase 1: Requirements

All proposed projects include 2 types of users: end users and implementers. End users are those individuals who use the intervention with the goal of changing their unhealthy lifestyle behaviors. Implementers are health care professionals who will administer and recommend the intervention to the end users. We will gather requirements from both types of users, specifically with respect to the content of the interventions and how the human-computer interaction should be designed.

#### Content Requirements

The content for each intervention will initially be based on the current best practice, gathered from scientific literature and experts. End users’ and implementers’ perspectives on the content of the interventions will then be explored through either focus group or individual interviews. An iterative process will be employed, which will allow content to be added, removed, or reworded in between interviews. Apart from the content itself, understandability and usability in terms of complexity of language and tone will be evaluated and adjusted for each project.

#### Interaction Requirements

Human-computer interaction and usability will be investigated through both heuristic evaluation [56] by interaction experts and usability tests with end users [57]. Several mock designs of the interventions will be produced, which will be analyzed by interaction experts according to a set of predefined heuristics (eg, a consistent design and error prevention). The mock designs will then be refined according to expert feedback. End users will then be asked to explore the mock designs without any guidance. While doing so, we will observe whether they are able to complete the goals that are given to them, for example, entering their daily alcohol consumption or finding information about how to deal with nicotine cravings. Refinement of the mock designs to remove obstacles that hinder the end users from completing the goals will be done in an iterative manner.

#### Phase 2: Development

The final content of the interventions will be decided by synthesizing the requirements gathered from phase 1. Behavior change technique analyses [58] will be conducted to elucidate how content connects to behavior change theory, for example, how motivation and self-regulation are addressed and supported by the content. The design of the interactive components that deliver the content of the interventions will be decided from the human-computer studies from phase 1.

The interventions will then be programmed and tested. Pilot studies will be conducted with end users to resolve technical issues and investigate how the final interventions are perceived. A questionnaire will be sent to pilot participants, with both fixed- and open-response options. Any major issues identified will be addressed before moving on to phase 3.

#### Phase 3: Evaluation

Randomized controlled trials (RCTs) will be conducted to explore the effects of the interventions in their respective target groups. The trial for each intervention aims to achieve the following:

1. Estimate the total effect of the intervention on individual lifestyle risk behaviors compared with a control setting.
2. Detect interactions among unhealthy lifestyle behavior changes (eg, those who stop smoking may also reduce their alcohol consumption).
3. Estimate to which degree the total effect is mediated through psychological factors (eg, self-efficacy and motivation).
4. Use baseline characteristics to predict who will benefit from the intervention.

For aims 1 and 2, regression models with appropriate distribution properties for the outcome measure will be used to estimate both the total effect of the intervention group compared with the control group on one lifestyle risk behavior and interaction effects among lifestyle risk behaviors. Regression coefficients’ significance will be assessed using null hypothesis significance testing and exploring Bayesian posterior distributions [59]. For aim 3, we will use causal inference to assess how interventional effects are mediated through sociopsychological factors [60]. For aim 4, we will use machine learning models that aim to predict whether a certain individual, given the individual’s baseline characteristics, will benefit from the intervention. This will allow us to identify subgroups that are not benefitting from the intervention, for which more research is required.

#### Outcome and Study Parameters

The primary outcome measures used in the RCTs will concern participants’ general lifestyle, in terms of alcohol consumption, physical activity, smoking, and diet. The method through which these outcomes are measured will differ slightly among projects with respect to feasibility. Secondary outcome measures will be project specific, for example, measuring complications after surgery and body weight. We will also measure sociopsychological characteristics such as self-efficacy and motivation as mediating factors.

#### Power Considerations and Study Sizes

In general, mHealth interventions have an effect on lifestyle corresponding to a standardized difference between outcomes in the intervention and control groups ranging from 0.20 to 0.35 (Cohen *d*) [17,61-67]. To identify the statistically significant differences of these magnitudes using two-sided *t* tests with 80% power at a 5% significance level, it will be necessary to recruit between approximately 150 and 400 participants per arm (separate power calculations will be conducted for each project).

#### Phase 4: Implementation

If effective, interventions will be implemented in routine practice, and the adoption of the interventions will be assessed by the Reach, Effectiveness, Adoption, Implementation, and Maintenance framework, which includes 5 elements [68]:

- *Reach*: The proportion of representative end users who engage with the intervention.
- *Efficacy*: The impact of the intervention on important outcomes.
- *Adoption*: The proportion of implementers who are willing to administer or recommend the intervention.
- *Implementation*: The consistency of delivery of the intervention in terms of implementers’ responsibilities and end users’ use of the intervention.
- *Maintenance*: To which extent the intervention becomes institutionalized and a part of routine practice.

Efficacy and long-term effects as part of maintenance will already have been assessed in phase 3. An initial estimation of reach will also have been done by comparing the number of invited participants, who are willing to partake, with the RCT.

To further investigate the reach, along with adoption, implementation, and maintenance, each project will assess the aspects of implementation in its respective health care unit. Implementers will be offered full access to administer the intervention. Adoption will be assessed through interviews, examining implementers’ willingness to administer and recommend the intervention. Implementation will be assessed by engagement over time by implementers as well as engagement of end users with the intervention itself. Finally, maintenance will be measured by interest from implementers to continue using the intervention as part of their routine care as well as through a questionnaire created around the normalization process theory coding framework on eHealth implementations [69].

### Project-Specific Details

The 7 main projects included in the MoBILE research program are described below.

#### Project 1: Pregnant Women

One project will extend ongoing research to develop mHealth tools to support a healthy lifestyle in pregnant women. We are currently evaluating the effect of an mHealth intervention (HealthyMoms app) on weight gain, diet, and physical activity during pregnancy in women who can speak and read Swedish well enough to benefit from the intervention [21]. We will extend this work by creating an mHealth solution (Healthy Migrant Moms) that is suitable for migrant women who do not understand Swedish well enough to use the original HealthyMoms app. Participants for the focus groups and panel evaluations will include both midwives and pregnant women recruited through the maternity clinic, Kvinnohälsan, Linköping. For the pregnant women, a series of focus group and individual interviews with different geographical and cultural backgrounds will be conducted with certified interpreters.

The subsequent RCT will follow a two-arm parallel-group design, for which participants will be recruited from the same maternity clinic. Both groups will receive standard antenatal care. However, the intervention group will also receive the novel intervention (Healthy Migrant Moms).

#### Project 2: Preschool Children

One project will implement a novel mHealth intervention [27], integrated into routine care, targeting parents of preschool children to promote the following main healthy behaviors in preschoolers: a healthy diet and physical activity. Participants for the focus groups and panel evaluations (ie, parents and nurses) will be recruited at the child health care in Östergötland. Part of this project includes translation and modification to also target priority populations such as children with a migrant background (approximately 25% of all children). Thus, focus group and individual interviews with parents will be conducted with both Swedish-speaking parents and with parents of different geographical and cultural backgrounds (using certified interpreters).

The subsequent RCT will follow a two-arm parallel-group design, for which participants will be recruited from child health care in Östergötland. Both groups will receive standard antenatal care. However, the intervention group will also receive the novel intervention.

#### Projects 3 and 4: High School and University Students

Two projects will develop mHealth interventions aimed at promoting improved healthy lifestyle behaviors among high school and university students. The interventions will be tailored to the two student groups based on input from the focus groups and panel evaluations.

Participants for the focus groups and interaction, as well as usability evaluations, will be recruited from high schools and student health care centers in Östergötland and from Linköping and Luleå University. A series of focus group and individual interviews with participants with different cultural backgrounds, of different age groups, and of different gender will be conducted.

An RCT for each student group will be conducted in collaboration with the student health care centers. The RCTs will follow a two-arm parallel-group design with an intervention group and a waiting-list control group. Participants will be recruited from high schools and universities located across Sweden.

#### Project 5: Individuals Seeking Help at Primary Health Care Centers

Healthy lifestyle promotion has been difficult to implement in routine primary health care. This project aims to develop an mHealth intervention targeting individuals seeking help at primary health care centers, for whom a lifestyle change is part of their treatment.

The focus groups and panel evaluations will comprise patient and health professional representatives from primary health care centers. We will conduct focus group and individual interviews with patients and health care professionals.

Participants for the RCT will be recruited from primary health care centers in Sweden. Health care professionals will recruit patients for whom they have determined that a lifestyle change should be part of their treatment. The trial will have a two-arm parallel-group design, in which the control group will be put on a waiting list.

#### Project 6: Patients in Clinical Care

One project will focus on self-care disease management and lifestyle risk behavior change among patients. The aim of the project is to enable the patients in clinical care to change their unhealthy lifestyle behaviors, monitor symptoms and signs from their condition, or learn more about self-management.

Focus groups and panel evaluations will be conducted, comprising both patients and professionals. The content of the intervention will therefore be tailored to each disease group.

An RCT will be conducted to assess the effect of the tailored intervention. Participants will be recruited from medical wards in Sweden. The trial will follow a two-arm parallel-group design, in which the control group will be put on a waiting list.

#### Project 7: Patients With Elective Surgery

This project will extend ongoing research on mHealth support for patients who need to quit smoking before surgery. The project aims to add support to the existing intervention for reducing alcohol consumption, increasing physical activity, and promoting a healthy diet.

Participants for the focus groups and panel evaluations will be solicited from surgical departments in Sweden. Focus group and individual interviews will be conducted with patients and health care professionals.

Surgical departments across Sweden will be the base for recruitment for the RCT, which will have a 2-arm single-blind design. The control setting will be treatment as usual, depending on the local routines in each department. Staff at the surgical departments will inform all patients with elective surgery about the study, and consenting participants will be able to sign up on their own by using their mobile phone.

## Results

**Figure 2:**
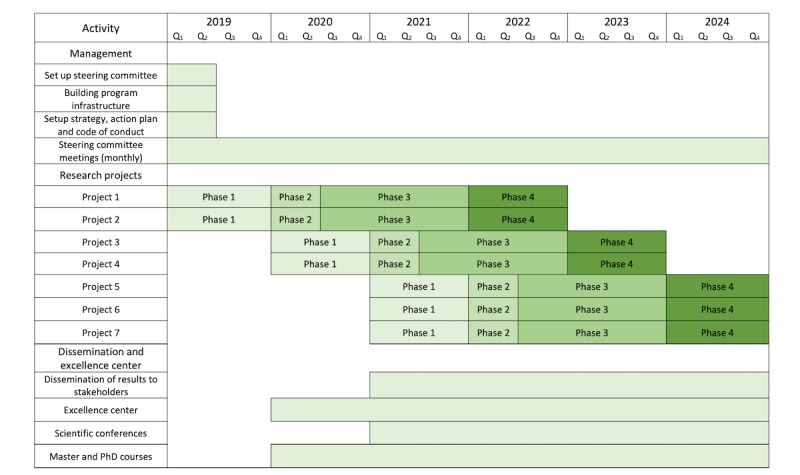
Time plan for the Mobile health Multiple lifestyle Behavior Interventions across the LifEspan research program. Phase 1: investigation of requirements by end users and implementers; phase 2: development of mobile health intervention; phase 3: randomized controlled mobile health trial; phase 4: implementation into practice.

The research program was funded in 2018 (FORTE, Dnr: 2018-01410, ML). The research program commenced in 2019 and is progressing according to the time plan presented in Figure 2. Briefly, the program will start with a short period where we will set up its infrastructure. Thereafter, the 7 projects (following the same 4 phases) will be executed. Projects involving pregnant women, preschool children, and high school and university students will be completed in the first 3 years, with the remaining projects being planned for the program’s final 3 years. Additional activities within the research program will include the following: dissemination of the results to the stakeholder, organizing scientific workshops and conferences, and organizing master’s and PhD courses on mHealth and lifestyle risk behavior topics. The first results will be available in 2020.

## Discussion

### Joining Resources

Currently, many public health and clinical researchers are interested in developing and disseminating mHealth tools to promote a healthy lifestyle. This is a positive development; however, it also raises some concerns regarding research and evidence gaps in the field. There is still much that we do not know when it comes to the effectiveness and dissemination of mHealth interventions. Before we can launch national campaigns promoting mHealth tools, we should invest time and resources in covering the knowledge gaps that we are currently facing.

The development of evidence-based digital tools is complex, as they should be guided by theoretical frameworks, and requires large interdisciplinary teams with competence in technology, behavioral science, and subject-specific areas (eg, nutrition and smoking cessation). Individual researchers or smaller research groups developing their own tools is not the way forward, as it means reinventing the wheel over and over again. The MoBILE research program therefore aims to join forces and learn from the past 10 years of mHealth research to maximize scientific outcomes, as well as the use of financial resources, to expand the growing body of evidence for mHealth lifestyle behavior interventions.

### Clinical Relevance and Limitations

The projects included in the research program aim to address multiple unhealthy lifestyle behaviors across the life span of different health care populations; thus, the potential health benefits from the program as a whole are wide reaching. For instance, smoking cessation among adolescents will reduce the burden of disease in the future, whereas reduced alcohol consumption can lessen the immediate risk of harm among those drinking and others in their proximity. In addition, as the MoBILE research program focuses on multiple lifestyle risk behaviors and as risks increase nonlinearly with the number of unhealthy lifestyle behaviors an individual presents, the health benefits from the interventions may be superior to previous interventions focusing on one lifestyle risk behavior. This is particularly important as data indicate that individuals often have multiple unhealthy lifestyle behaviors [70].

The MoBILE research program focuses on identifying at-risk individuals from specific health care contexts, such as maternity clinics or primary health care services. This limits the reach of the included interventions and excludes a wider audience not captured within the defined contexts of the program. From a public health perspective, we wish to engage with as many at-risk individuals as possible; thus, our intention is to learn about mHealth multiple lifestyle behavior interventions within the health care context and then expand our research to include the general population.

## Appendix

Multimedia Appendix 1 Grant proposal reviewer report from Forte - Forskningsrådet för hälsa, arbetsliv och välfärd.

## Abbreviations

eHealth: electronic health
IMPACT: innovative use of mobile phones to promote physical activity and nutrition across the lifespan
LiiR: Lifestyle Intervention Implementation Research
mHealth: mobile health
MoBILE: Mobile health Multiple lifestyle Behavior Interventions across the LifEspan
NCD: noncommunicable disease
RCT: randomized controlled trial
WHO: World Health Organization
