# Supplementary material for: The Mobile Health Multiple Lifestyle Behavior Interventions Across the Lifespan (MoBILE) Research Program: Protocol for Development, Evaluation, and Implementation
Source: JMIR Res Protoc. 2020 Apr 20;9(4):e14894. doi: 10.2196/14894 (PMC7199135; doi:10.2196/14894)
Supplement: Multimedia Appendix 1 [file resprot_v9i4e14894_app1.pdf]

FORTE: Forskningsrådet för hälsa, arbetsliv och välfärd

# *Beredningsgruppens yttrande*

| 2018-01410 | Marie Löf | Beredningsgrupp: Vård18 |
|---|---|---|

**Utlysningsnamn:** Vårdforskning 2018

**Projekttitel (svenska):** mHealth Multiple Lifestyle Behaviors- livsstilsinterventioner via mobiltelefoner inom hälso-och sjukvården till olika patientgrupper av barn och vuxna

**Bidragsform:** Programbidrag

**Sökt inriktning:** Vård

## Inledning

### En kort inledning som beskriver syftet med projektet

This application looks to provide mobile-enabled support for lifestyle change in relation to physical activity, diet, smoking, alcohol use and other areas for people with T2DM and other chronic conditions, bringing together two strong research groups.The team seeks to use the same theoretical framework for different patient groups with a view to generating an efficient, high-quality process for developing and evaluating eHealth.

## Bedömning

### Forskningsprogrammets vetenskapliga kvalitet (originalitet, teorianknytning samt relation till tidigare forskning inom området och den internationella forskningsfronten)

The programme offers a highly relevant set of research questions that seem timely and tie in well to Swedish government ambitions around the potential of digital health. The Panel welcomed the team's ambition to bring together experts around mHealth to avoid reinvention of the wheel, their use of relevant theoretical frameworks, and their plan to use a common framework adapted to different types of audience, e.g. pregnant women. The commitment to transparency in use and description of the phases of development and implementation outlined in the proposal was also welcomed.

### Studiedesign, metoder och datamaterial

Overall the methods, materials and study design are well accounted for. One minor concern for the Panel was that the RCTs for each intervention be sufficiently resourced to produce valid and reliable results that would merit publication and inform subsequent implementation or further development work.

### Programmets vetenskapliga värde

The team make a good case for the scientific value of the programme, and use high-quality and appropriate methods to achieve this.

### Resultatens förväntade praktiska betydelse/relevans

The creation of a national research centre of excellence bringing together these researchers and others was seen as an important component of the proposal. The team seemed to have considered carefully the resourcing needs for this work, and how this relates to capacity development of researchers at different stages in their careers.

### Samhällsrelevans

The programme is highly relevant to the needs of society, and the applicants make a good case for the potential of mobile technologies in addressing lifestyle-related health. Work on creating interventions that address multiple risk factors simultaneously was seen as particularly important.

### Genus- och mångfaldsperspektiv i forskningens innehåll

Careful consideration is given in the proposal to the needs of different groups, including pregnant women and those from socioeconomically disadvantaged and migrant backgrounds. This is well justified in terms of the relative health status and needs of these groups.

---

### Programmets genomförbarhet

The panel agreed that the programme looked credible and feasible.

---

### Programledarens och forskargruppens forskningskompetens

The programme brings together two research groups with clearly relevant experience, and a range of important expertise, including various healthcare disciplines, psychology, computer science and others. The involvement of international experts is also welcome.

---

### Personal och budget

The Panel felt this was a strong team with a good track record of research in this area.

---

### Sammanfattande bedömning

In all the Panel were impressed by this programme, and saw it as having important potential value and a carefully considered timeline and potential for impact.

---

## Etikprövning

### Bedöms sakna etisk problematik eller behov av etikprövning finns

The Panel agreed that appropriate ethical review would be required for this programme.

## Förslag till beslut

### Bevilja, bevilja i mån av medel eller avslå

Approve
